# Molecular Markers of Blood Cell Populations Can Help Estimate Aging of the Immune System

**DOI:** 10.3390/ijms24065708

**Published:** 2023-03-16

**Authors:** Natalia Rybtsova, Tatiana N. Berezina, Stanislav Rybtsov

**Affiliations:** 1 Institute for Regeneration and Repair, Centre for Regenerative Medicine, University of Edinburgh, Edinburgh EH16 4UU, UK; 2 Department of Scientific Basis of Extreme Psychology, Moscow State University of Psychology and Education, 127051 Moscow, Russia; 3 Centre for Cell Technology and Immunology, Sirius University of Science and Technology, Sirius, 354340 Sochi, Russia

**Keywords:** aging, aging biomarkers, biological age cellular immunity, inflammation, immune senescence, virus resistance, immune system

## Abstract

Aging of the immune system involves functional changes in individual cell populations, in hematopoietic tissues and at the systemic level. They are mediated by factors produced by circulating cells, niche cells, and at the systemic level. Age-related alterations in the microenvironment of the bone marrow and thymus cause a decrease in the production of naive immune cells and functional immunodeficiencies. Another result of aging and reduced tissue immune surveillance is the accumulation of senescent cells. Some viral infections deplete adaptive immune cells, increasing the risk of autoimmune and immunodeficiency conditions, leading to a general degradation in the specificity and effectiveness of the immune system in old age. During the COVID-19 pandemic, the state-of-the-art application of mass spectrometry, multichannel flow cytometry, and single-cell genetic analysis have provided vast data on the mechanisms of aging of the immune system. These data require systematic analysis and functional verification. In addition, the prediction of age-related complications is a priority task of modern medicine in the context of the increase in the aged population and the risk of premature death during epidemics. In this review, based on the latest data, we discuss the mechanisms of immune aging and highlight some cellular markers as indicators of age-related immune disbalance that increase the risk of senile diseases and infectious complications.

## 1. Introduction

Immunity is an indispensable defence system distributed throughout all organs and tissues to perform important nonredundant functions. The main functions are protection against infections, tissue regeneration and remodelling, removal of senescent and tumour cells, and scavenger function [1]. The aging process is accompanied by changes in the immune system, increasing its dysfunction and imbalance. Undoubtedly, the main function of immunity is to protect against infections and prevent premature mortality, which determines the average life expectancy. In developing countries, 30 to 70% of the population still dies of infections before reaching old age. Yet, in the aging population in developed countries, the incidence of age-related imbalances or dysfunctions of the immune system increases, raising the risk of developing autoimmune, inflammatory, and oncological disorders. The frequency of premature deaths from infections or infectious complications grows with age, which is especially dramatic in epidemics [2].

Thus, it is extremely important to identify hidden vulnerabilities of the immune system to protect risk groups during epidemics and social upheavals and to maintain working capacity and health in old age. Circulating blood cells, resident hematopoietic cells in tissues, and plasma signaling proteins contribute significantly to aging. Studies on heterochromic transfusion of plasma or blood in animals of various ages have identified cell populations and factors involved in the ageing process [3,4,5,6].

This review summarizes recent studies on the analysis of the aging of immune cell populations. Assessment of early cellular indicators of aging and markers of immune system vulnerability is essential for diagnosing and analyzing the risks of premature disability in the elderly. Considering that infectious complications, the frequency of autoimmune disorders, and malignant neoplasms are closely related to age, this review describes cellular indicators of immune system aging, which can warn about the risk of susceptibility to dangerous diseases. The correlation between ageing and immunodeficiency states is outlined. Health monitoring indicators are considered for the timely inclusion of rehabilitation programs to maintain working capacity in middle and pre-retirement age.

## 2. The Immune System and Environmental Survival

A healthy immune system is certainly a critical factor in survival in the natural environment. Humanity, having provided itself with safe comfortable living conditions, healthy food, clean water and medical care, has significantly increased average life expectancy; however, the example of the recent COVID-19 epidemic once again showed that life can end due to problems with an ageing immune system [7,8,9].

The role of the immune system in survival is unambiguously illustrated in the analysis of immunodeficiency mutations in the human population and in transgenic animals. The severe combined immunodeficiency SCID mutation blocks the maturation of T lymphocytes and, depending on the type of mutation, leads to the death of children due to primitive infections during the first two years of life. The stay of such patients in sterile conditions prolongs their life, but the risk of developing cancer remains high. Prior to the onset of infection, treatment with bone marrow transplantation increases five-year survival in 80–95% of children with the SCID mutation [10].

Animal studies have shown that additional mutations that block the proper development of other cellular components of the immune system caused even more severe consequences. In addition to the SCID mutation, NOD/SCID/IL-2Rγ (NSG) mice have a knockout in Interleukin-2 receptor gamma (IL-2Rγ), which prevents the development of all components of adaptive immunity. Furthermore, NOD genetic background significantly weakens the innate immunity of NSGs reducing complement-dependent haemolytic activity. These mice die within two weeks in an infectious environment. Even when kept under sterile conditions, lifespan is reduced by more than 30% (~20 months) compared to C57BL/6 wild-type mice (~30 months). Both inflammatory and neoplastic conditions contribute to morbidity and mortality in aged NSG mice [11]. Interestingly, NOD/SCID (NOD/ShiJic-Prkdc^scid^) mice, which, unlike NSG, have fully functional IL-2Rγ and retain remnants of NK cell activity, but live even shorter lives. On average, they live up to 8.5 months and die from spontaneous thymus lymphomas. The last two examples illustrate the importance of maintenance of immune homeostasis [12].

It is also dangerous to disrupt the regulatory (suppressor) function of the immune system. Mutation in the Foxp3 gene, which is critical for the development of a suppressor population of T regulatory cells (T_regs_), causes multi-organ autoimmunity and death of children in the first two years after birth [13].

Thus, the immune system can not only prevent premature death from infections, but, in case of its imbalance, can cause inflammation, and autoimmune disorders and, thus, trigger critical conditions and accelerate the development of senile diseases, reducing the life expectancy of animals and humans.

Research has also shown gender dimorphism in the aging of the immune system. The immune system: the effectiveness of adaptive immunity in men quickly declines in comparison to women after 65. The authors discuss the contribution of gender factors to the longer life expectancy of women [14]. Sex differences in the aging of the primate immune system have also been observed [15,16].

Ageing of several cellular components of immunity results in gradual failures in the functioning of this protective mechanism and in various immunodeficiencies that cause impaired immune surveillance and reduction of certain peripheral immune responses [17]. A detailed analysis of the quantitative characteristics of the main populations of the immune system and their molecular markers outline pathological processes leading to dangerous complications. The review of recent publications on single-cell RNA transcriptomic and multichannel flow cytometry, as well as the analysis of functional changes in immune populations performed in this review provide a basis for the development of methods for monitoring and predicting age-related diseases and require further statistical and clinical verification.

### 2.1. α/β T Cells

**Figure 1:**
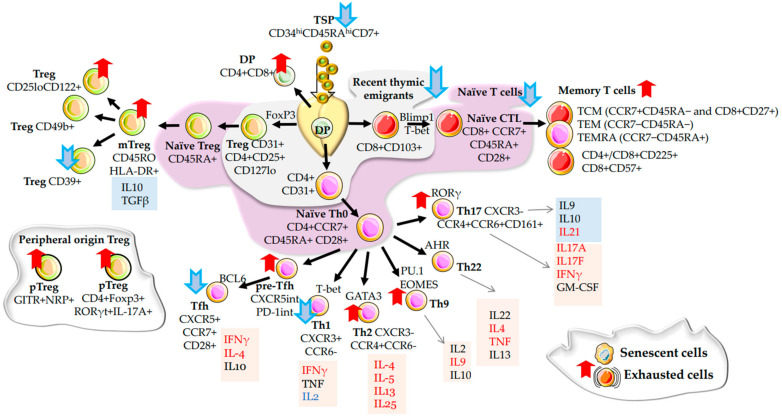
Ageing of T-lymphocytes. The migration of thymus seeding progenitors (TSP) from the bone marrow is demonstrated at the top of the graph (age-related migration decline is shown by a blue arrow pointing down). In the thymus (yellow organ), the double positive (DP) population matures into different T-lymphocytes branches, including cytotoxic lymphocytes: CTL (cell nucleus shown in the red); T-helpers (Tfh, Th1, Th2, Th9, Th22, Th17) (purple nucleus); and T-regulatory cells (brown nucleus). The age-related migration of the DP population from the thymus (cell with a green nucleus) due to thymic involution is indicated on the top left. The grey area near the thymus shows all cells’ recent thymic emigrants and the purple area shows naïve T-lymphocytes decreasing in number with age (blue arrows show a decrease with age). At the top right are T-cell-memory populations elevated with age (red arrow pointing up). The dynamics of ageing of other populations are described in the text. The blue and red arrows demonstrate the direction of the shift of the specifically designated populations during ageing. The orange background indicates pro-inflammatory cytokines and the blue background shows anti-inflammatory cytokines secreted by the indicated T-cell populations. The red font shows cytokines that increase with age, while the blue font shows those that decrease with age. Near the black arrows (they indicate the direction of development), transcription factors are shown that determine the development of particular T-lymphocytes lineage. The bottom left shows T_regs_ formed in peripheral tissues. The lower right shows senescent and exhausted T-lymphocytes increasing in number with age.

α/β T cells are an important tool in the adaptive immune system. T cells mature in the thymus and carry out a basic set of adaptive immune responses against intracellular parasites such as viruses, bacteria, and protozoa. According to their immune functions, T cells are divided into CD3+CD8+ cytotoxic lymphocytes (CTL)–they deliver cytotoxic function against cells with intracellular infections and CD3+CD4+ T-helper cells (Th) can regulate the direction of the immune response. Depending on external signals, naïve, not yet committed, Th0 develop into Th1 (CXCR3+CCR6−), which responds to intracellular pathogens and tumour cells by cytokine production. In Th2 (CXCR3−CCR4+CCR6−), which initiates the immune response to extracellular pathogens, they stimulate humoral reactions (production antibodies by B-cells). CXCR3−CCR4+CCR6+CD161+ Th17 cells, play an important role both in initiating tissue inflammation and activating neutrophils to fight extracellular infections [18]. Finally, in Th9, they cause mucosal inflammation [19] and fight tumours [20] (Figure 1). Other subtypes are regulatory T cells (T_regs_) discussed in Section 2.2. and follicular T helper cells (Tfh) in Section 2.4.

The T cell receptor (TCR) is the main instrument of T lymphocytes for recognizing intracellular antigens of pathogens presented in the context of a major histocompatibility complex (MHC). The number of diverse antigen recognition repertoires is determined by the number of precursors coming from the bone marrow, the efficiency of V(D)J recombination, and subsequent positive and negative selection in the thymus. TCR β repertoire diversity dwindles among effector T cells in old age. TCR-β diversity reduced from approximately 20 million different TCR-β chains in young age to 200,000 in the elderly after 70 [21]. The number of naïve T cells also reduces in line with the decline of TCR β diversity [22,23].

Single-cell transcriptome analysis shows that the number of terminally differentiated memory, effector, and exhausted cells increases, while the number of naive cells decreases. The signs of T cell ageing are a decrease in the population of naive α/β T cells positive for CD28 and CD45RA markers, and an expansion of memory cells with CD45RO and CD57 surface markers [23]. Recognizing the importance of naïve α/β T cells for immune system function, various studies have identified populations with classical and complementary marker sets that refine the signs of immune system ageing. For example, the number of naive T cells (CD45RA+CD45RO−CD62L+CCR7+) circulating in the blood is reduced with age. A particularly significant decrease in the number of naïve CD4+CD31+ and CD8+ CD103+ T cells (recent emigrants from the thymus) is also observed. [24]. An assessment of recent thymic emigrant cells by the number of T-cell receptor excision circles (TREC) also showed a significant decline with age [25]. The ratio of naïve (CD45RA+CD28+CCR7+) CD4+ and CD8+ T cells was higher at a younger age but decreased with age, while the proportion of T central memory TCM (CCR7+CD45RA− and CD8+CD27+), T central memory TEM (CCR7−CD45RA−), and TEMRA (CCR7−CD45RA+) cells increased [26,27,28]. Cytometry by the time of flight (CyTOF) and scRNAseq analyses confirm a decrease of both naïve CD4+ CCR7^hi^CD69^lo^ T cells and naïve CD8+ CCR7+ T cells in a healthy old adult. [29]. In brief, the age-dependent decrease in the number of naive cells is associated with a decline in thymopoiesis due to age-related thymic and reduced production of lymphoid precursors of bone marrow origin.

The CD4+ Th/CD8+ cytotoxic T cell (CTL) ratio is considered a useful indicator of immune system health [30]. During ageing, the number of CD4+ cells decreases, while the number of CD8+ T cells increases [23,28]. At a young age, the CD4+/CD8+ ratio fluctuates around 2, while, in the elderly, the ratio becomes reversed (<1) [30]. In addition, an analysis of the old Swedish population (86–92 years old) revealed several blood parameters that are associated with reduced two-year survival: poor T-cell proliferative response, low CD4/CD8 ratio, and low CD19 percentage [31].

Another comprehensive study demonstrated a quantitative decrease in all CD3+ cells, both CD4+ and CD8+, in the elderly. A particularly strong decline was observed in the naive CD8+CD95− a population with simultaneous colonial dominance of CD28− T cells and NK cells with an inflammatory phenotype. Most of these cells were incapable of dividing and had shortened telomeres. The inflammatory profile in both Th1 and Th2 helpers was also enhanced [32]. In recent studies using scRNAseq, the inflammatory nature of T cells has also been shown by increased expression of HLA−DRB5, PDCD5 and PSMA2 molecules [23].

The number of CD4+CD8+ (double positive population, DP) and proliferating T cells were increased in the blood of elderly people compared to young individuals [23]. The DP population is associated with inflammatory, autoimmune disorders, and cancer. The study on primates also showed an increase in the DP population with age. The single-cell analysis also observed significant heterogeneity in the DP population. Twelve different cell types have been identified, which have been suggested to have both peripheral and thymic origins. For example, T cells with the phenotype CD31+CD4+CD8+ were found to be similar in phenotype with recent thymic emigrants. It is assumed that the DP population grows in the bloodstream due to background inflammation and thymic involution [33,34].

The elevation of several differentiated CD8+ T cell populations in blood was indicated in the elderly, including GZMK+ (Granzyme K) effector memory, GZMB+ GNLY+ (Granzyme B, Granulysin) cytotoxic T cells (CTL), and PDCD1+ (PD-1) exhausted T cells. The number of CCR6+ effector memory CD4+ Th cells only slightly increases with age [23]. Perhaps the reduced number of naïve T cells is one of the main reasons for poor resistance to novel viral infections. Another reason is reducing responsiveness and production of IL-2–the main cytokine driving T helper and T regulatory cells to proliferation. The several differentiated inflammatory cytokine-producing T cells increase their number, with age, e.g., Th1 cells producing IFN-γ and Th2-secreting cytokines IL-4 and IL-10 [35]. It was also noted that, in old age, there is a gradual shift in the balance from the Th1-type to the Th2-type of the immune response [36].

More and more authors associate age-related inflammation with an accumulation of Th17 cells. Th17 cells are characterized by the expression of transcription factors RORγT and surface receptors (CD161, CCR4, CCR6) [37]. For example, the number of Th17 cells is elevated due to periodontal disease, one of the risk factors for cardiovascular disease and other deadly inflammatory complications [38]. Indeed, Th17 cell induces inflammation by release of IL-6, IL-17A, IL-17F, IL-21, and IL-23. This set of cytokines is associated with the development of the age-related inflammatory pattern. Administration of metformin reduces Th17 cytokine secretion resulting in inflammation decline in the elderly [39]. The serum level of IL-21 in PBMCs increases with age, evidently due to local inflammation of the epithelium, which possibly reflects compensatory anti-inflammatory mechanisms [40].

Recently, an additional subgroup of T helper cells, called Th22, has been characterized. Differentiation into Th22 is triggered by contact with TNF and IL-6, and by stimulation with a specific antigen. During differentiation, the release of IL-22 increases. This interleukin regulates the secretion of antimicrobial proteins which protect and regenerate tissues. IL-22 performs a nonredundant function, protecting the skin and epithelial tissues. Th22 differentiation depends on the aryl hydrocarbon receptor (AHR) transcription factor. The small molecule VAF347 is an AHR agonist that selectively directs Th cells differentiation into IL-22-secreting cells [41]. The dual role of IL-22 in the activation of antimicrobial defences and in tissue regeneration was investigated by modifying the IL-22 protein, revealing differences in signalling by various mutants either through the IL10β chain or through the IL-22Rα chain on the receptor complex [42].

In addition, an age-related increase in T cells with interferon-inducible transmembrane molecule CD225 (IFITM) on the surface has been recently shown [43]. CD225 is localized both on the cell surface and in the cellular plasma on early lysosomal membranes of CD4+ T helper and CD8+ T cells and transposed on the surface upon stimulation by anti-CD3/CD28 antibodies. CD225 molecules almost do not express on naïve T cells. Upon TCR ligation and co-stimulation CD225+ T cells acquire cellular resistance to viral infection [44]. Ablation of CD225 facilitates efficient differentiation to Th1 immune response and resistance to Th2-dependent immune pathology, including asthma, and Th17-dependent inflammation in colitis [45,46].

Recent studies have strongly indicated a decrease in the plasticity of naive T-helper cells in the elderly. At a young age, they can differentiate depending on the required T-cell response, while in the elderly, in addition to differentiation into Th17, naïve CD4+ T cells after stimulation are strongly inclined to differentiate into Th9 cells secreting IL-9—factor of mucosal inflammation. Th9 cells are upregulating the TGFβR3 receptor, transcription factors PU1, and EOMES. Tumour-specific Th9 cells are highly effective for adoptive cancer therapy. Interestingly, most cancer checkpoint inhibitor molecules are downregulated in Th9 cells [47]. Comparative analysis of naïve Th9 CD4+ cells showed a significant increase in the old individuals activating factors BATF, IRF4, and a decrease in the inhibitory factors BCL6 and ID3, while young individuals had high levels of FoxO1 and Stat6 expression in Th9 cells [48]. The contribution of Th9 cells and IL-9 to the manifestations of inflammatory bowel syndrome and other mucosal inflammation have been recently discussed [19].

The gene ontology and pathway analysis for the aged T cell population display upregulation of several pathways, including inflammatory (TNF, IL-1 signalling, adaptive immune response) and the apoptotic signalling pathway [23]. Intrinsic signs of ageing consist of an increase in intracellular transcriptional variability between individual Th cells of the same population [49] and disorganized chromatin modifications of human immune cells [50].

In brief, the hallmarks of ageing in α/β T cells are a decrease in CD4+ and an increase in CD8+ cells number. The ratio of these two parameters determines human health and the state of the immune system. There is a decline in the population of naïve T cells carrying CD95−, CD28+, CD45RA+, CD45RO−, CD62L+, and CCR7+ markers, including recent thymic emigrants CD4+CD31+ and CD8+ CD103+ cells. On the contrary, the number of memory cells bearing CD45RO and CD57 markers on the surface increases (Figure 1). Dominant clones begin to prevail in the circulation, which significantly affects the recognition repertoires of the TCR receptors. Also noted is the accumulation of CD28− PD1+ cells with age, which are not capable of proliferation, have shortened telomeres and are considered senescent cells.

The lesion In T-helper differentiation plasticity results in Th9, Th17 bias, and CD225+ cell number increase. Single cells RNA sequencing (scRNAseq) showed upregulation of TNF, IL-1 and IFNγ signalling pathways, HLA-DRB5, PDCD5, and PSMA2 molecules in the elderly. All these data indicate an expansion of T cells with an inflamed phenotype. In addition, the amplitude of the response to inflammatory stimuli drops, and the instability of gene expression rises.

Therefore, finding ways for thymus rejuvenation [51], overcoming T cells senescent [52] and exhaustion [53], maintaining T-helper plasticity and maintaining the production of naive thymocyte progenitors in the bone marrow is an extremely important scientific task for translation into preclinical and medical studies. New approaches have to be developed to preserve the health of the population, especially considering the risk of epidemics of viral infections [54].

### 2.2. Regulatory T Cells

Regulatory T cells (T_regs_) are crucial adaptive immune cells for immunoregulation by keeping a check on its over activation. The main T_regs_ population is defined by surface markers as CD3+CD4+CD25+CD127^lo^ and expression of the master regulator of T_regs_ Forkhead transcription factor (Foxp3) [55,56,57]. Foxp3-deficient mice exhibit lethal autoimmunity and lack of CD4+CD25+ cells [55]. In line with this, a mutation in the Foxp3 gene in humans disrupts immunotolerance and causes immune dysregulation, polyendocrinopathy, enteropathy, an X-linked (IPEX) disease characterized by multi-organ autoimmunity. Most affected children die within the first two years of life [13]. Knockout of IL-10, the main anti-inflammatory factor produced by T_regs_ cells, leads to cell senescence, premature development of mouse frailty, impaired immunotolerance mechanisms, and reduced mice lifespan [58]. These data demonstrate the significant role of regulatory T cells in immune system function and healthy longevity. [59].

As is the case with other T cells, the balance between CD25+CD45RA+ “naive-T_regs_” and CD25^hi^CD45RO+ “memory T_regs_” phenotype shifts with age [60] (Figure 1).

**Figure 2:**
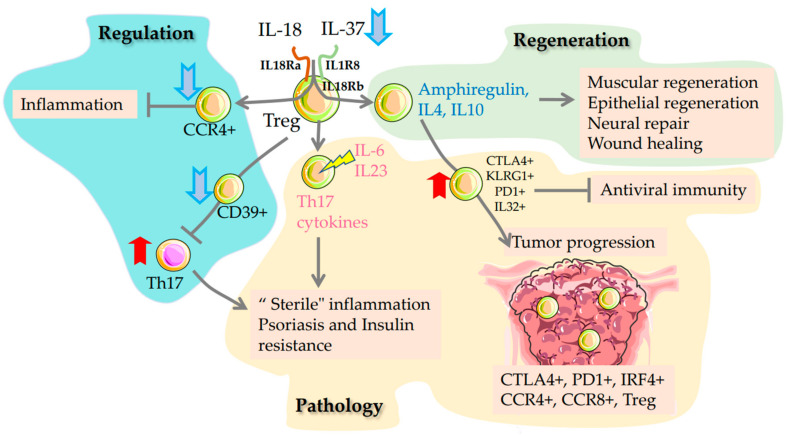
Ageing of T-regulatory cells. T_regs_ ageing mechanisms change the functional properties of cells. The immunosuppressive (regulatory) function is reduced due to decreased maturation and differentiation of T_regs_ populations (blue area). Shift in the balance between IL-18 and IL-37 alters the efficiency of T_regs_ differentiation and their ability to support tissue regeneration (green area). The cytokines involved in regeneration are shown in blue.

Regulatory T cells also express on the surface several molecules important for their function, including GITR, neuropilin-1(NRP1), CD62L, CTLA-4, and PD-1. Ageing is accompanied by a decline in thymus function. Accordingly, the amount of tT_regs_ (of thymic origin) is reduced in circulation compared to peripherally induced pT_regs_ with age [61]. The accumulation of pT_regs_ with age may be one of the reasons for ineffective suppression of the peripheral immune response, increased inflammation, and tumour cells escape from immune surveillance. Moreover, the age-dependent rise in IL-6 levels and the shift in the balance of Th17/T_regs_ cells may play a key role in tissue “sterile” inflammation. [62]. As shown earlier, CD39+ T_regs_ cells are implicated in suppressing Th17 cells, which take part in type 2 diabetes development (Figure 2). Hence, CD39+ T_regs_ cell number is decreased in type 2 diabetes patients [63]. Selective removal of resident T_regs_ cells from fat using injections of anti-ST2 antibodies normalizes insulin sensitivity of adipose tissue and reduces overall inflammation [64]. Apparently, various populations of T_regs_ cells have either a protective or a pathological role in metabolic diseases. T_regs_ of peripheral origin have recently been shown to have significant plasticity and, when stimulated by IL-23, are able to upregulate Th17 cytokines. These cells with the CD4+Foxp3+RORγt+IL-17A+ phenotype themselves support the inflammatory response in psoriasis [65].

Accumulation with the age of T_regs_ with a certain phenotype is one of the causes of pathologies (yellow area). T_regs_ secreting IL-32 can, on the one hand, block antiviral immunity and, on the other hand, protect tumours from immune surveillance. In the tumour (lower right), T_regs_ cells are shown carrying molecules that block antitumor immunity.

Pro-inflammatory background and accumulation of inflammatory factors, i.e., IL-6, IL-23, induce differentiation into T_regs_ secreting pro-inflammatory cytokines of the Th17 phenotype. Regulatory T cells are shown with a brown nucleus. The increase in T_regs_ populations with age is shown by the red arrow pointing up, and the decrease by the blue arrow pointing down.

The accumulation of T_regs_ cells with age affects lifespan in different ways. Clinical data revealed that more CCR4+ cells in circulation were associated with longer survival in older donors. Notable, CCR4+ T_regs_ (functionally primed cells) exhibit suppressive function regardless of T-cell receptor co-stimulation, compared to non-primed CCR4-negative T_regs_. [66]. In old mice, the level of IL-2 in the circulation decreases. This may be why CD25^lo^(IL-2Rα) T_regs_ with a high level of expression of CD122 (beta chain of IL-2 and IL-15 receptor) significantly accumulate in circulation. The authors hypothesized that, in older mice, T_regs_ are desensitized to IL-2 and increased reliance on IL-15 for their survival [67].

T_regs_ are also involved in the regenerative function of the immune system. It was recently suggested that two interleukins IL-37 and IL-18 play a key role in turning on the reparative or immunosuppressive function of T_regs_. After IL-18 stimulation, T_regs_ produce amphiregulin, which triggers multiple regenerative actions [68,69], (Figure 2). Produced by epithelial cells, IL-18 is involved in the regulation of the protective function of T_regs_ to prevent colitis [70]. Amphiregulin and IL-10 are involved in muscular regeneration [71], epithelial regeneration [72] neural repair [73], and wound healing [74]. However, their role in the development of tumours has also been shown [75]. Recent studies revealed that amphiregulin is produced by senescent cells and its presence is a negative background provoking expression of programmed cell death 1 ligand (PD-L1) and cancer development [76,77], (Figure 2). This role of T_regs_ associated with age-dependent carcinogenesis is being actively investigated. IL-18 has also been shown to be involved in inflammatory processes. IL-37 and IL-18 share a common receptor chain, IL-18Ra, signalling using IL-1R8 or IL-18Ra light chains, respectively. Thus, the regulation of immunosuppression and tissue regeneration is a subtle mechanism involving both ligands. Interestingly, only IL-37 decreases in circulation with ageing [78].

Age-related decline in expressions of the DCAF1/GSTP1/ROS pathway reduces the suppressor function of T_regs_ and imbalances T_regs_ and conventional T cells, increasing inflammation and senescence. The study also discussed the role of the classical pathway associated with mTOR in T_regs_ metabolism. The authors proposed mTOR as a target for reducing the number of senescent T_regs_ cells [79]

Some severe viral infections can be associated with accumulation in the circulation of T_regs_ secreting IL-32 and carrying CTLA-4, KLRG1 and PD1 on the surface [80]. Such T_regs_ suppress the antiviral response against COVID-19 and are involved in blocking cancer immune surveillance [81,82]. Moreover, numerous clinical studies have shown that blocking T_regs_-specific immune checkpoint molecules CTLA4, and PD1, removing CCR8+ T_regs_ or from tumour enhances antitumor immunity [81,83,84]. A deletion exclusively in T_regs_ Interferon Regulatory Factor 4 (IRF4) delayed tumour growth in mice. Moreover, tumour infiltration of large numbers of IRF4+ T_regs_ is associated with poor prognosis in patients with various types of human cancer [85], (Figure 2). It is likely that age-related accumulation in the circulation of T_regs_ with the phenotypes described above weakens tissue immune control over inflammation and cancer [59].

Thus, T_regs_ cells play a key role in immunity, peripheral tolerance, prevention of autoimmune diseases, and limitation of chronic inflammatory diseases, thereby slowing down the development of age-related diseases. In connection with the age-related thymic involution, there is a decrease in naive T_regs_ CD4+CD25+CD45RA+ and an increase in CD4+CD25^hi^CD45RO+ “T_regs_ memory” cell number. Hence, the amount of T_regs_ originating from the thymus reduces, and the number of T cells of peripheral origin positive for the GITR markers, NRP1, increases (Figure 1). Peripheral T_regs_ are unstable and can change their function depending on the microcirculation. The number of CD39+ T_regs_ that control Th17 cells also decreases. CCR4+ T_regs_ have a TCR-independent suppressor function age-dependent drop in CCR4+ cell number is associated with negative outcomes and elevation of this T_regs_ amount with a positive prognosis in the elderly (Figure 2). Impaired suppressor function is considered a key trigger for age-related inflammatory and oncological diseases.

### 2.3. γ/δ T Cells

γ/δT cells are an important minor subset of all T cells in the lymphoid organs and in the blood. However, in tissue analysis, they make up a much larger percentage of the T cell population. γ/δT cells go through all stages of development in the thymus, representing an alternative population of T cells that play a substantial role in the control of body tissues, such as skin, gut, lungs and more. For example, most mucosal intraepithelial lymphocytes are γ/δT cells that are essential for peripheral tolerance. They also have a regenerative function, contributing to tissue repair and healing of injuries, and are also involved in tissue homeostasis and in the surveillance of infection [86]. In addition, they can recognize internal antigens such as phospholipids, cells that have lost normal glycosylation, and other signs of tumorigenesis—inflamed cells activated by rapid division, tissue compression, or necrosis. These abnormities activate γ/δT cells and secretion of inflammatory cytokines without stimulation in the context of MHC. This activates γ/δT cells and secretion of inflammatory cytokines without stimulation in the context of MHC [87]. Since γ/δT cells are not restricted to MHC-mediated antigen presentation, they become very popular for application for anticancer therapy [88].

The total number of γ/δT cells decreases with age twice. However, the number of CD28+ γ/δ T cells remains at the same level during ageing, while the amount of CD27+ γ/δ T cells reduces with age by more than 30%. [28]. There was also a decline in the function of these cells with age. γ/δT cells gradually lose their ability to be activated in response to IL-12, IL-15, and IL-18 in the elderly [89]. Memory-like CD57+Vδ1 T-cells that represent one of the major Vδ T-cells subsets in the circulation accumulate with age while the number of CD57+ Vδ2 T-cells stays stable during all life [90].

The decrease in the number of γ/δT cells with age is one of the risk factors for cancer in the elderly. The development of new methods for activating and stimulating the proliferation of specific γ/δ T cell populations is a promising strategy for maintaining healthy ageing. [87].

The age-related dynamics of γ/δT cells in the blood and in lymphoid organs do not fully reflect the real events of ageing. Without analysis of γ/δT cells in peripheral tissues, the picture remains incomplete. Resolving the difficulties of such analysis will bring a deeper understanding of their true significance for the organism and the influence of the dynamics of their ageing on the development of age-related diseases.

### 2.4. B-Cells

**Figure 3:**
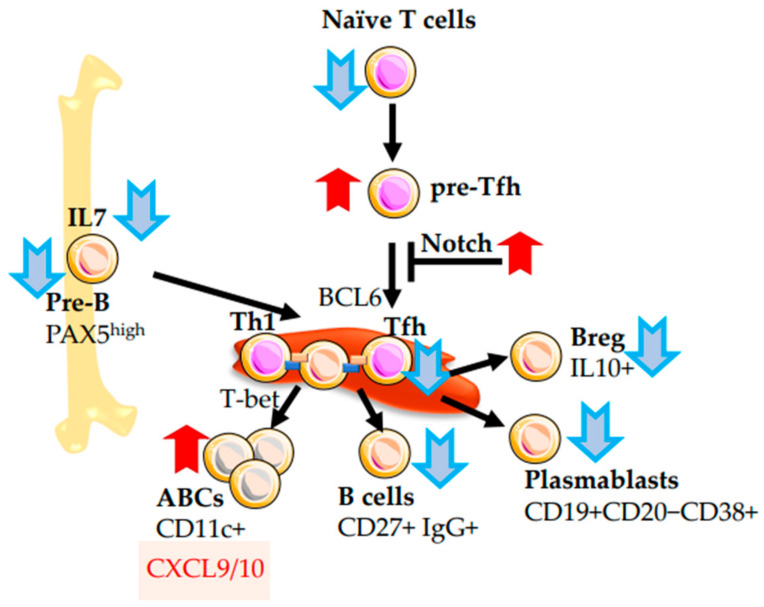
Age-related changes in the B cell development. A delay in Tfh cell development reduces the production of mature B-cells in the spleen (brown). Production of immature B-progenitors is also reduced due to a decrease in systemic expression of IL7 by the thymus and in bone marrow (yellow bone). In blood circulation, the number of Pax5+ cells is also reduced. While the production of ABC-producing proinflammatory cytokines (CXCL9/10) is dramatically increased with age, which is one of the risk factors for inflammatory disorders. The increase in B-cells populations with age is shown by the red arrow pointing up, and the decrease is shown by the blue arrow pointing down.

B cells are an integral part of the adaptive immune system that fights extracellular and cavitary infections. B cells produce antibodies specific to pathogens, and present antigens in the MHC context for T cells. Production of high-affinity antibodies in response to immunization decreases with age. Accordingly, the response to the vaccine weakens with age. One reason is the general decline in the number of mature B cells [32]. Another reason is a decrease in the number of multipotent common lymphoid progenitors that affect the successive development of pro-B cells, pre-B cells, and, ultimately, the number of immature B cells in the bone marrow [91]. Progenitor maturation is impaired with age due to the reduced ability of pro-B cells to respond to IL-7 and a decrease in IL-7 secretion by niche cells in the bone marrow [92,93]. In addition, in the ageing organism, the number of precursors expressing PAX5 decreases in the bone marrow (Figure 3). This regulatory factor determines the correct specification of B-cells at the stage of development of pro-B cells in the bone marrow. Additionally, the number of B cells with low PAX5 expression in the bloodstream of the elderly increases, accompanied by the accumulation of CD10-CD20+CD21−CD27 atypical memory B cells and CD21−T-bet+CD11c+ age-associated B cells [94].

Immature B cells emerge from the bone marrow and, after migrating to secondary lymphoid organs, complete maturation into two major peripheral subsets takes place: marginal zone B cells and follicular B cells that determine the correct structure of the lymphoid organs [95]. Various subsets of B cells perform unique functions in the human body and contribute to the balance and effectiveness of the immune response. A decrease in the production of high-affinity antibodies occurs due to a reduction in the influx of B-cells into the lymphoid organs and due to ageing-related changes in other components affecting the structural organization of the spleen and lymph nodes [96]. The immune response to vaccination or infection requires the combined efforts of several types of immune cells and the proper organization of lymphoid organs. The generation of long-term humoral immunity occurs in the lymphoid organs during the formation of the germinal canters (GC), where B cells, CD4+ follicular helper T cells, stromal cells, follicular dendritic cells, and macrophages work together to produce high-affinity secreting antibodies, plasma cells, and B-memory cells [97].

As mentioned above, the age-related involution of the thymus restricts naïve T-cells production. In addition, with age, some T-helper cells lose their ability to penetrate the lymph nodes and initiate the formation of germinal centres (GCs), which is a critical step for the maturation of B cells. [98]. A decline of naïve T-cells penetration limits the cross-presentation of antigens during the development of B cells GCs in the spleen and lymph nodes and, therefore, limits the ability to produce high-affinity antibodies after immune stimulation. A reduction of Tfh number in peripheral blood was observed in elderly people after the influenza vaccination, and the production of vaccine-specific antibodies was five times lower than in young people. [97]. It was found that CD4+CXCR5^int^PD-1^lo/int^ precursors of follicular T-helpers (pre-Tfh) accumulate in old mice and humans after immunization. Pre-Tfh differentiation occurs independently of IL-12 and TGFβ and is controlled via the Notch pathway [99]. Apparently, impaired maturation of Tfh cells (CD4+CXCR5^hi^PD-1^hi^) affects their migration to GC and the maturation of high-affinity antibodies (Figure 3). However, the predictable rise with age in the expression of IFN-γ, IL-17, and IL-4 in Tfh cells is partly responsible for the increase in age-related inflammation and Th2-biased immune response [40]. After immunization, the production of low-affinity autoreactive antibodies is increased in old mice compared to young animals. The same process could be one of the reasons for the increase in autoimmune disorders in humans with age [100]. Probably, the concentration of autoreactive low-affinity antibodies after immunization can be a reliable indicator of ageing.

In old adult humans, in both memory B cells (CD19+CD27+IgHG1+) and Naïve B cells (CD124+(IL-4R) IgHD+), expressions of JUNB, IGHA1, SSR4, and CXCR4 are increased, indicating Th2 type humoral response bias and reducing anti-viral responses. In Naive B cells, cytokine-mediated signalling pathways are also stimulated with age [23].

A decrease in the concentration of immunoglobulins in blood plasma has been detected in older adults [101]. In older people, there is a depletion of the antibody repertoire and the accumulation of a limited population of memory B cells. Moreover, the absolute number and the frequency of CD27+ memory circulating B cells are reduced in elderly humans. The total number of CD27− naïve B cells number is slightly increased, and they acquire higher resistance to apoptosis [102]. Recent studies have shown that the number of CD19+CD20−CD38+ plasmablasts is significantly reduced in aged individuals, which definitely contributes to the decrease in antibody production with age. [26]. In old adults, Plasmablasts/plasma cells, or so-called antibody-secreting cells, also express a high level of immunoglobulin genes MZB1 and a subset of ITGAX+ B cells. The authors defined them as age-associated B cells [23].

T-bet is known to be involved in the regulation of autoantibody production [103]. As mentioned above, age-associated B cells (ABCs) have a CD19+ IgM+ CD11b+ CD11c+ phenotype, and as shown in previous studies, ABCs express T-bet. Many ABCs especially accumulate in the spleen and in the peripheral blood of old female mice. ABCs are associated with autoimmune diseases more prevalent in females [104,105,106]. The expansion of atypical memory B cells (CD10−CD20+CD21−CD27−) [94] and the accumulation of CD95^hi^CD21^lo^CD11c+T-bet+ late memory B-cells with a senescent expression profile were also noted [107]. ABCs CD19+IgD–CD27−CD21^lo^ (memory-like B cells) producing autoreactive antibodies were also described in aged women with systemic lupus erythematosus [108].

T-bet+ antigen-specific Th1 cells can stimulate memory B cells via CD40L and IFNγ, promoting the generation of CXCL9/10-producing ABC-like B cells presumably involved in inflammatory senile diseases such as rheumatoid arthritis [109] (Figure 3).

Secretion of CXCL9/10, a cytokine, attracts immune cells to the site of infection. CXCL9 levels begin to rise sharply after the age of 60, which is associated with the inflammatory ageing of the organism [110]. CXCL9 is also implicated in the pathology of cardiovascular disease; however, its role in antitumor immunity has also been documented [111,112]. CXCL9, in addition to ABSs, is also secreted by endothelial cells and tumour-associated macrophages. An increase in the level of CXCL9 with age in circulation reduces the ability of endothelial cells to form microvascular networks, and the ability of vessels to contract, which causes pathologies of the vascular system [110].

Regulatory B cells (B_regs_) can suppress the immune response by secreting interleukin-10 (IL-10). B_regs_ cells have a heterogeneous phenotype and have recently been shown to produce IL-6 and TNF in inflammatory conditions, along with anti-inflammatory IL-10 expressions. However, when developing immunotolerance to liver transplants, B_regs_ can express only IL-10 [113]. Similarly, spontaneously tolerant renal transplant recipients exhibited a rise in the number of CD38+CD24+IgD+ B cells, some of which express IL-10 [114]. The pronounced increase in inflammation with age is partly due to the decline in B_regs_ numbers. As shown recently, pro-inflammatory TNF-secreting B cells persist in the elderly, while the number of IL-10-secreting B cells drops. The authors also associated the age-related decline in IL-10+ B cells with an increase in rheumatoid factors and anti-nuclear antibodies in the elderly [115]. Thus, B-cells undergo significant changes with age. There is an increase in the number of CD19+CD27− and CD19+CD124+IgHD+ naïve B-cells in the blood and, vice versa, a decrease in the memory cells of CD19+CD27+IgHG1+ and CD19+CD20−CD38+ plasmablasts, which reduce the systemic production of high-affinity antibodies. The migration of Tfh cells in the lymphoid organs is impaired in part due to compromised maturation of pre-Tfh that accumulate in the circulation. Furthermore, there is the age-associated accumulation of CD19+IgM+CD11b+CD11c+ (T-bet+) B cells as well as a decrease in IL-10+Breg. The number of senescent B cells increases. All these changes reduce the response to vaccination and the production of high-affinity antibodies in the elderly and increase the risk of developing autoimmune and inflammatory diseases associated with age.

### 2.5. NK-Cells

Natural Killer cells (NK) are part of the innate immune system. NK cells are the first line of defence, performing a cytotoxic function against tumours and virus-infected cells. The high cytotoxic activity of NK cells is associated with healthy ageing and longevity. While low cytotoxicity of NK cells is associated with inflammatory diseases, predisposition to atherosclerosis, low resistance to fungal infections, risk of complications and mortality from infectious diseases, and poor response to vaccinations [116,117,118].

Using scRNAseq and subsequent flow cytometry validation, it was shown that the cytokine-producing NK population (CD3−CD56^bright^CD16^lo^) is reduced with age while the cytotoxic NK population (CD3−CD56^dim^CD57^lo^) and terminally differentiated NK (CD3−CD56^dim^CD57+) cells are expanded. Thus, NK cells exhibit an age-related increase in the CD56^dim^/CD56^bright^ ratio. In addition to this, the apoptotic signature was increased in NK cells, which was expressed in the upregulation of DDIT4, ISG20, and CASP4 genes, and downregulation of DDX17, PCBP1, and TRIM56 expressions. These gene patterns are also enriched in Th2 cellular responses to lipopolysaccharides, along with the weakening of antiviral responses [23]. Another study using multichannel cytometry in a large sample of patients of different ages showed a rise in both CD16^lo^CD57+CD56^bright^ and CD56^dim^ populations with age, while CD16+CD57− tends to decrease [26].

With age, NK cells become rich in inhibitory receptors. A significant correlation has been found between the level of the inhibitory molecule KIR (killer inhibitory receptor) and ageing. On the contrary, a marked age-related decrease in the expression of NKG2A and KLRG-1 was registered [119]. As a result of such changes, the function of NK cells is impaired and resistance to viral, bacterial, and fungal infections reduces, while nonspecific inflammatory response rises [120]. However, it is necessary to consider the presence of chronic infections that stimulate an increase in NKG2A expression regardless of age. For example, this was indicated for cytomegalovirus infection [121].

Thus, with age, there is a decrease in the number of NK cells producing cytokines and an increase in the number of cytotoxic cells and terminally differentiated NK cells. Moreover, scRNAseq showed an increase in the genetic profile of the Th2 immune response, which is a sign of impaired NK cells’ response to cells affected by viruses and malignant cells. Along with a general rise in the expression of inhibitory molecules on the surface of most of the NK cells, age-related changes indicate a decline in the cytotoxic response to bacterial and fungal infections.

### 2.6. Dendritic Cells

Dendritic cells (DCs), along with macrophages, represent the main population of professional antigen-presenting cells in the innate immune system. DCs, despite their scarcity in the blood, play a major role in peripheral tissues by capturing antigens and migrating to secondary lymphoid organs for their presentation into T cells. Two major subsets of dendritic cells have been found in human blood and tissues: CD14−CD16−CD11c+ myeloid DCs (mDCs) and CD14−CD16−CD11c−CD123+ plasmacytoid DCs (pDCs) [97]. There has been a general decrease in the number of DCs in adults and elderly individuals. The greatest reduction was observed in the number of CD123+ pDC [26].

**Figure 4:**
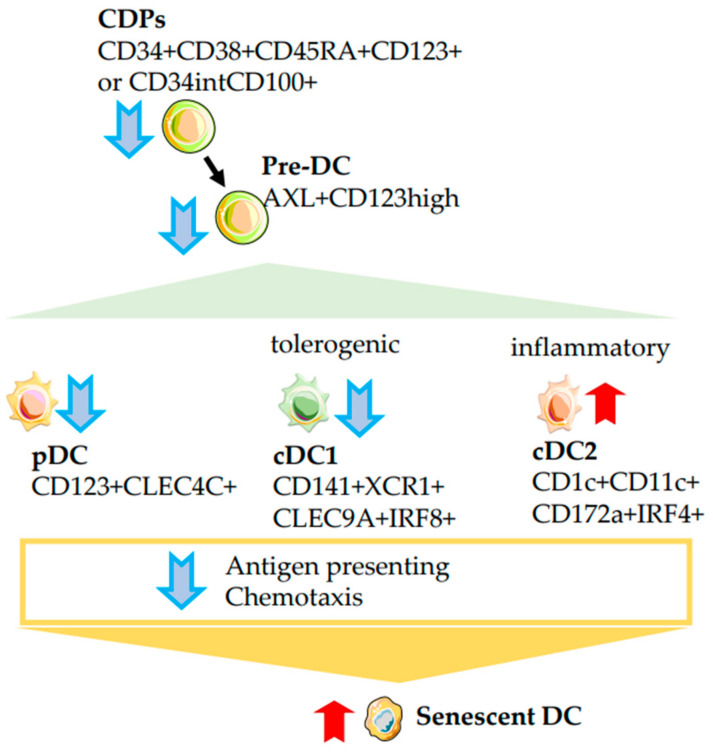
Ageing of dendritic cells. The decrease in the number of common dendritic cell progenitors (CDPs) and immediate dendritic cell precursors (pre-DCs) affects the development of the entire DC hierarchy. The age-related decrease in the number of DC precursors reduces the total number of DCs, as a result, antigen presentation, migration to inflammation foci and lymphoid organs become insufficient. The balance between tolerogenic and inflammatory DCs changes with age. Plasmacytoid pDCs, tolerogenic conventional DC1s (cDC1s), decrease in number, while inflammatory DC2s increase. The rise in DC populations with age is shown by the red arrow pointing up, and the decline is shown by the blue arrow pointing down. A gradual accumulation of aging DCs is observed.

The most complete functional classification of DCs has been developed relatively recently. The term ‘conventional dendritic cells (cDCs)’ refers to all DCs other than plasmacytoid DCs. They are conditionally divided into two subtypes, DC1 and DC2. It has been shown that cDC1s are considered tolerogenic cells and can even induce tolerance in inflammatory cDC2s [122,123] (Figure 4). The analysis of these subtle newly discovered subsets of cDCs showed that the inflammatory CD1c+CD11c+CD172a+IRF4+ cDC2 population increases with age. Whereas tolerogenic CD141+XCR1+CLEC9A+IRF8+ cDC1s, CD123+CLEC4C+ pDCs, AXL+CD123^hi^(pre-DC) and common dendritic cell progenitors (CDPs) CD34+CD38+CD45RA+CD123+ or CD34^int^CD100+ all decreased in old adult. Furthermore, accumulation of dysfunctional senescent dendritic cells is observed in aged adults [23,26,124,125]. The study also shows an age-dependent impairment of dendritic cell maturation, their ability to effectively present antigens, and migration to lymphoid organs [126].

Other subsets of dendritic cells (DC3, iDC), recently identified and revised using single cell analysis, require further study of their function and contribution to immune system ageing [125].

Thus, with age, there is an increase in inflammatory cDC2 and a decrease in tolerogenic cDC1, pDCs and their precursors, as well as a reduction in their ability to respond to stimuli, present antigens, and migrate to secondary lymphoid tissues.

### 2.7. Monocytes (MC)

Monocytes are a population of innate myeloid cells circulating in the blood. They play major roles in the defence against pathogens (inflammation regulation, phagocytosis, and antigen presentation), homeostasis, and tissue repair. Circulating monocytes are traditionally divided into three phenotypic types. The classical CD14+CD16− monocytes (cMC) carry the pattern-recognizing and chemokine receptors, e.g., CCR2, which directs them to the foci of inflammation. While settling in tissues, MCs differentiate into resident macrophages and release antimicrobial proteins (myeloperoxidase, lysozyme C) and reactive oxygen species (ROS). They also phagocytize and present on their surface antigens of pathogens and cell debris [127]. In addition, they may mediate tissue repair and remodelling functions, including wound healing and angiogenesis [128]. CCR2+ cMC makes an important contribution to combating SARS-CoV-2 and limiting airway inflammation in transgenic mouse models [129].

Another type is CD14+CD16+ “intermediate-like” monocytes (intMC). They differentiate in tissue-resident macrophages and express class-II MHC on the surface. In response to TLR4 stimulation, they begin to actively phagocytize and produce high amounts of both pro- or anti-inflammatory cytokines [126].

The third type is CD14^lo^CD16+CX3CR1+ non-classical monocytes (ncMC). They differentiate into macrophages that perform Fc receptor-mediated phagocytosis. Some of these macrophages “patrol” the walls of blood vessels, checking them for damage.

**Figure 5:**
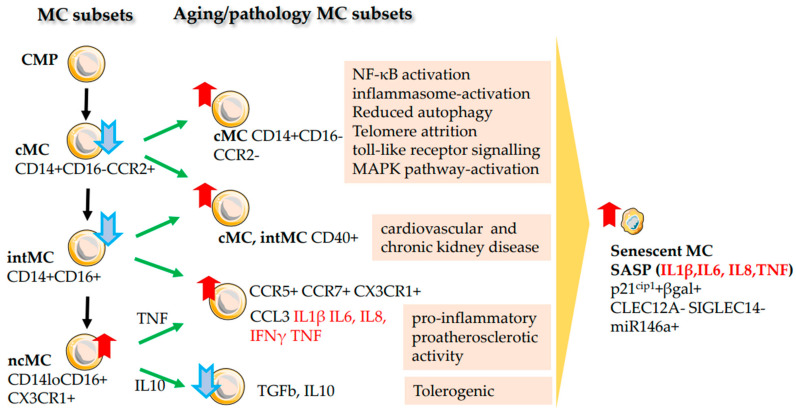
Ageing of Monocytes. The development of monocytes (MCs) from a common myeloid progenitor (CMP) is shown (see MC subsets). As a result of ageing, the number of classical (cMC) and intermediate MC (intMC) decreases, while the number of non-classical Monocytes (ncMC) with inflammatory profile increases. The accumulation of subsets of MC and the pathological consequences associated with aging are demonstrated on the right. For ageing intMCs and ncMCs, changes in the expression of additional cytokine receptors and pro-inflammatory factors (in red) are shown (see Aging MC subsets). The direction of MC development (black arrows) and possible ways of MC ageing (green arrows) are indicated. The increase in MC populations (red arrow pointing up), and the decrease (blue arrow pointing down) are shown. Age-related accumulation of senescent MCs (red arrow), senescence-associated secretory phenotype (SASP) and typical markers of senescent MCs are demonstrated on the right.

In response to IL-10, they can differentiate into anti-inflammatory macrophages. Upon encountering viral components or immune complexes containing nucleic acids, ncMS can differentiate into pro-inflammatory macrophages secreting TNF, IL-1β, IL-6 and CCL3 [130] (Figure 5).

A significant increase in the number of CD14+CD16− and CD14+CD16+ monocytes in circulation in the elderly was found. Experiments have shown that MCs have shorter telomeres, produce IL-6, TNF, IL-1β, and carry chemokine receptors (CCR2, CCR5, CCR7, CX3CR1) on their surface, i.e., they have a pronounced pro-inflammatory phenotype and pro-atherosclerotic activity [131].

More recent studies have revealed that the number of intermediate monocytes (CD14+CD16+) significantly decreases with age [26], contrary to other reports [131,132]. About a double increase in the frequency of CD14+CD16− cMC and CD14^lo^CD16+ ncMC is observed. These inflammatory monocytes in elderly individuals were enriched for IL-1β, TNF, IL-8 expression and IFN-γ—signalling signature [26].

CD40+ expressing cMCs and intMCs are induced in cardiovascular disease and chronic kidney disease progression [133]. Age-specific cells express the cell division arrest marker CDKN1A (p21^cip1/waf1^), genes that regulate the prevention of apoptosis and suppressors of DNA damage repair.

Aged MCs reduce the expression of cell adhesion molecules CLEC12A and SIGLEC14, which serve cell-cell communication during inflammation and innate immune response [23]. Monocytes in the elderly respond poorly to stimuli and are unable to secrete proinflammatory cytokines. For example, accumulated with age, CD14^lo^CD16+ ncMCs express a high level of senescence marker miR-146a (a negative regulator of the TLR pathway). The accumulation of these ncMCs and increased activity of NF-κB correlate with the production of IL-1α, TNF, and IL-8/CXCL8 [134]. Older cMCs (CD14^hi^CD16−) appear to be significantly affected regardless of their significant expansion in old age. They activate an inflammatory signature (NF-κB signalling, inflammasome-activating toll-like receptor signalling, and MAPK metabolic pathway). Additionally, old cMCs reduce autophagy, vesicle-mediated transport, and RNA splicing. All these results point to an age-related decline in phagocytosis, antigen presentation, and other signs of ageing [23]. With age, there is a decrease in the number of CCR2+ phagocytic myeloid cells, the expression of markers of phagocytosis (e.g., CD68 when activated), and the expression of a number of functional markers on MCs, including CX3C motif chemokine receptor 1 (CX3CR1) and CD62L, which all affect antigen presentation, MC adhesion, and migration to foci of inflammation [132,135].

Proinflammatory cytokines (TNF, IL-1β IL-6) are increased in frail elderly people. This condition stimulates the expansion of CD11b+CD15+CD33+HLA-DR− myeloid-derived suppressor cells (MDSC). These cells were found elevated significantly in elderly donors with a history of cancer. The authors discuss the contribution of MDSCs in age-dependent elevation cancer incidence [136].

Thus, the number of monocytes with inflammatory phenotype rises and senescent macrophages accumulate in ageing tissues, remaining in an inflamed, activated state. These macrophages express high levels of CD38, the main consumer of NAD+. High consumption of NAD+ reduces its concentration in tissues and leads to tissue starvation and dysfunction [137]. Such ageing MC/macrophages are practically incapable of phagocytosis and fulfil an antigen-presenting function.

### 2.8. Granulocytes/Neutrophils

Granulocytes/Neutrophils are the abundant circulating blood leukocytes involved in phagocytosis and they are able to release inflammatory cytokines. The lifespan of granulocytes is several days and their pool is maintained by the constant generation of new cells from precursors in the bone marrow. Neutrophils are involved in the development of chronic inflammatory diseases, and cancer, and exhibit an ageing immunity phenotype [138]. In the elderly, a negative correlation has been shown between the number of phagocytosed bacteria per neutrophil cell and age [139]. In another study, it was shown that granulocytes in the elderly have an impaired ability for chemotaxis, degranulation, and phagocytosis [140].

Granulocytes are the first to come to the sites of inflammation and, through degranulation, they release active enzymes and active antibacterial molecules that perform anti-infective functions. CD64 (FCγR1) is upregulated on granulocytes and monocytes within 4–6 h after contact with interferon gamma and GM-CSF. This is an important indicator of inflammation. A CD64 marker can help distinguish infectious and autoimmune inflammation [141]. Recently, the level of expression of CD64 on neutrophils was proposed as an important indicator of acute peritonitis in children and the elderly [141,142].

The induction of CD64 on granulocytes and neutrophils plays an important role in phagocytosis, which explains its functional significance for the analysis of inflammaging and other types of “sterile” inflammation [143].

The decrease in the activity of granulocytes and neutrophils is one of the important reasons for the decline in the rate of healing of wound lesions. Due to the low activity of these cells, bacterial films formed on the surface of the wound prevent healing [144]. As mentioned above, the ratio of myeloid and lymphoid precursors changes with age, which reduces resistance to infectious diseases. If the NLR-index (neutrophil-to-lymphocyte ratio) exceeded 3.1, then the risk of going into a critical condition in patients with COVID-19 infection increased. In patients older than 50 years, the NLR index influenced the severity of the symptoms, regardless of other factors [145].

### 2.9. Eosinophils

Eosinophils (EOs)-granular cells play the main role in the fight against parasitic invasions. However, their role in allergic, asthmatic, and other pathologies has also been shown. In humans, they are determined using scattering properties on flow cytometry along with expression of CD125 (IL-5 R alpha), CCR3, and Siglec-8 in humans or Siglec-F in mice. EOs are also positive for CD11b and EMR1 [146]. Recently, numerous receptors have been found on the surface of eosinophils that switch their functions, allowing them to participate both in immune reactions and in the regulation of homeostasis [147]. Analysis of eosinophils from the blood circulation showed that degranulation in response to interleukin-5 was significantly reduced in elderly donors. Other functions did not differ from young donors [148]. However, their most important and indispensable function is eosinophils fulfilled in tissues, where they are involved in many important processes and are also the causes of age-related pathologies.

Adipose tissue eosinophils (ATE) are important cells that control the development of susceptibility to age-related diseases. Located in adipose tissues, inflammatory polarized ATEs are involved in obesity-associated inflammation and metabolic disorders. Accumulating in tissues with age, ATEs provide the body with inflammatory factors, contributing to the growth of tissue inflammation. At the same time, parabiotic models show the possibility of restoring the normal ATE profile using the serum of young animals. Serum analysis showed that the factor IL-4, derived from ATE, plays an important role in this recovery [146].

Thus, the role of eosinophils in the development of brown fat, as well as in the polarization of M2 macrophages by the secretion of IL-4, has been shown. However, this study also shows the negative role of IL-4 in adipose tissue inflammation and the development of glucose tolerance [146,149,150,151]

Eosinophils are involved in thermoregulation by modulating adipose tissues, so under the influence of cold temperatures, white adipose tissues through FGF21—β-Klotho signalling activate CCL11 and recruit eosinophils to participate in thermoregulation through Th2 immune response [152].

Another important function of eosinophils in the immune response is the clearance of apoptotic T cells in the thymus. The decline in this function with age contributes significantly to thymic involution. [153]. In the lungs, eosinophils function as antigen-presenting cells. In addition to class-II MHC, they express costimulatory molecules CD40, CD80, and CD86, activating Th2 responses [154]. By secreting cytokines eosinophils can switch the immune response. So, when activated, they are able to secrete both Th1 cytokines (IFN-γ, IL-8) and Th2 cytokines (IL-4, IL-13) [155], and also participate in the immunotolerance reaction by secreting IL-10, TGF-β [156,157].

Eosinophils are actively involved in regenerative processes. Thus, by releasing IL-4 and IL-13 in damaged muscle tissue, eosinophils activate adipocyte-fibroblast precursors, stimulating them to myogenic differentiation [158]. Secretion of IL-4 causes hepatocyte proliferation and regeneration of the liver [159] and thymus [160]. Eosinophils are involved in vascular remodelling by stimulating angiogenesis by secreting osteopontin [161]. Moreover, the overactivated of reparative eosinophil can cause increased tissue remodelling in pathological esophagitis and asthma [162].

A series of interesting experiments have recently been carried out, where it was shown that transplantation of eosinophils from young animals can rejuvenate the adipose tissues of older animals and improve regeneration and the state of the vascular system [163]. Eosinophil transplantation had also a rejuvenating effect on the immune system, through an improved response to vaccination in older mice [146].

Thus, eosinophils play a significant role in the ageing of body tissues, participating in the inflammatory process. Identification of eosinophils in tissues is difficult for routine diagnosis. However, according to the results of numerous studies discussed here, the inflammatory profile of eosinophils increases with age, and the effectiveness of their functions decreases [23].

## 3. Conclusions and Future Perspectives

Several key processes occur during the aging of the immune system: a decrease in the total volume of the bone marrow due to its replacement with fatty tissues; disruption of the precursors production by bone marrow stem cells following by a decrease in the number of both thymocyte precursors and precursors of B cells; thymic involution reduces release of naïve lymphocyte in blood circulation; depletion of T and B -cell receptors repertoire; the number of fully functional cells of the immune system with the necessary plasticity (e.g., T-helpers) are reduced; infraction of the maturation of cells involved in the production of high affinity antibodies; disruption of structure of secondary lymphoid organs; accumulation of exhausted lymphocytes unable to proliferate and react on stimuli; accumulation of terminally differentiated cells of adaptive immunity and an increase in the number of senescent cells secreting several pro-inflammatory cytokines; the elevation of the inflammatory background of the body; offense in balance of the regulation between the suppressor function, and inflammatory reaction, an excessive tolerance in some peripheral tissues. Besides, the function of scavengers is significantly reduced: phagocytosis, chemotaxis to the sites of inflammation, the ability to degranulate in response to infectious stimuli and plasticity of myeloid cells. All these processes lead to a decrease in adaptive and innate immunity in response to infections, a reduction in the ability to control the amplitude of the immune response, which slows down the cleansing of tissues from ageing and damaged cells and the maintenance of body tissues in a functional state. What generally reduces the effectiveness of immune reactions against infectious and oncological diseases reduces the body’s immune surveillance.

The biological age of the individual is determined by the physiological state of the body, muscle tone, and resistance to environmental stresses. The basic factor affecting biological aging is the status of the immune system which is involved in the cleansing and regeneration of tissues and protecting against aggressive infections. The weakening or exhaustion of some parts of the immune system with age has a significant impact on the whole organism increasing the biological age. The imbalance of immunity, an increase in the background of inflammation, local immunotolerance, and a decrease in regenerative and scavenger functions are the main signs of the immune system ageing.

In the last few decades, several approaches developed to provide an overall picture of immune aging and predisposition to age-related diseases. Among those are monitoring of inflammatory factors [110], analysis of phagocytic, antigen-presenting and reparation activity [164], identification of markers of senescent populations [52,137,165], analysis of the dynamics of immune cells, and the study of the immunotolerance of individual cell populations. Tracking the accumulation of ageing cells and terminally differentiated cell populations, analysis of a decrease in the diversity and specificity of the receptors of B- and T cells, as well as the identification of dominant clones of immune populations are discussed as important approaches for preclinical and clinical studies of age-related pathologies.

Studies of markers of cell populations are especially important for testing drugs, new and traditional natural substances for slowing down ageing, and for preventing senile diseases [166,167,168]. Depletion, damage, and transition to a senescent state are associated with age-associated accumulation of mutations and DNA modifications. Therefore, special attention is paid to drugs protecting DNA damage or drugs enhancing DNA reparation [169,170,171], and to studies of drugs that reduce the activity of retroviruses and retrotranspozones in the elderly [172,173].

Overall, all the cell indicators described above provide an objective picture of immune system ageing and develop monitoring approaches and methods of targeted action in the personalized medicine of the future. Blocking factors that negatively affect health and life expectancy, the use of senolytics, senomorphics, substances enhancing DNA reparation, inhibitors of transposases, and reverse transcriptases could be the reasonable strategy for preventing early disability and extending active life in pre-retirement and retirement age.
